# Viral Myositis Secondary to Influenza A in a Preschool Child in Saudi Arabia: A Case Report

**DOI:** 10.7759/cureus.53179

**Published:** 2024-01-29

**Authors:** Rawia F Albar, Rahaf A Hubayni, Raghad A Aldahhas, Elaf I Khshwry

**Affiliations:** 1 Pediatrics, King Abdulaziz Medical City, Ministry of National Gaurd, Jeddah, SAU; 2 Medicine, College of Medicine, King Saud Bin Abdulaziz University for Health Sciences, Jeddah, SAU

**Keywords:** ck, pediatric, upper respiratory tract infection, influenza a virus, viral myositis

## Abstract

Infective myositis is a rare complication of viral infection, occurring most commonly in children. Here, we present the first case report in Saudi Arabia that describes a four-year-old healthy female who presented to the emergency department with a history of fever associated with coryzal symptoms for four days and a one-day history of bilateral lower limb pain and an inability to walk without assistance. Lower limb pain was not associated with joint pain, swelling, or skin rashes. The respiratory virus panel was positive for influenza A, and she was found to have increased levels of creatine kinase (CK). The patient was diagnosed with viral myositis secondary to influenza type A infection and was admitted for dehydration. She was treated successfully with supportive measures and oseltamivir. The patient's condition improved three days after the initial presentation and was discharged and followed up to ensure resolution. Extensive laboratory assessment and hospitalization can often be deemed unnecessary, given that the majority of cases of viral myositis carry a positive prognosis and are self-limiting. Therefore, it is important to consider viral myositis as a potential diagnosis for a child presenting with difficulties walking, particularly if these symptoms arise following a respiratory infection.

## Introduction

Infective myositis is a type of inflammatory myopathy characterized by inflammation of the skeletal muscles [1]. This condition frequently occurs in school-age children, with a median age of onset at 8.3 years, following a respiratory tract infection [2,3]. In terms of gender distribution, males are twice as likely as females to be affected, with a ratio of 2:1 [4,5]. Infective myositis can be attributed to various pathogens, with influenza types A and B being among the most common causative agents [1]. In the United States, myositis is more common in cases of influenza B than A, with rates of 33.9% and 5.5%, respectively. Infective myositis can also be caused by coxsackie, parainfluenza, adenovirus, enterovirus, human T-cell leukemia-lymphoma virus, hepatitis B and C, and even SARS-coronavirus. Myositis has a low incidence, ranging from 0.23 to 2.6 cases per 100,000 children [6,7]. In a retrospective study carried out at King Fahd Hospital of the University in the Eastern Province of Saudi Arabia, the prevalence of myositis was estimated at 3.17 per 100,000 in pediatric patients under 12 years old. The study mentioned the absence of Saudi studies to compare their findings with. However, no virology study was conducted for any of the patients in this research [8].

Clinical manifestations of infective myositis typically involve the sudden onset of muscle pain, gait abnormalities, swelling, and tenderness, primarily affecting the gastrocnemius and soleus muscles [1]. These symptoms usually manifest three days after the resolution of the initial viral illness and are preceded by prodromal symptoms of viral infection, such as fever, malaise, cough, sore throat, and rhinorrhea [1]. Distinguishing this condition from more severe causes of walking difficulties is accomplished by assessing the presence of lower limb tenderness, normal muscle strength, and intact tendon reflexes [9].

Recommended laboratory tests for diagnosing infective myositis include creatine kinase (CK), liver function tests, a full blood count, urine myoglobin, and viral studies [2]. In cases of infective myositis, elevated CK levels are often accompanied by increased lactate dehydrogenase (LDH) and aminotransferase values [2]. Full blood count results may reveal leukopenia, neutropenia, and lymphocytopenia [2,3]. Although the infection is typically self-limiting, antiviral medications can be administered to control respiratory and systemic complications [1]. The prognosis for viral myositis is generally excellent, with complete clinical and laboratory recovery expected within two weeks [3]. Despite its generally good prognosis, myositis is commonly overlooked in the search for other differential diagnoses of acute-onset weakness [6]. Therefore, it is important to include infective myositis in the differential diagnosis of acute weakness and calf pain [6]. We present the first case report of viral myositis secondary to influenza A in Saudi Arabia and discuss the differential diagnosis for the patient.

## Case presentation

A four-year-old female, who is medically and surgically free, presented to the ED with bilateral lower limb pain and an inability to walk without assistance for the past day, along with one episode of vomiting. She also experienced a subjective fever, a runny nose, a sore throat, and a mild cough. The patient was in good health until four days prior to the ED visit, when she developed a subjective fever and coryzal symptoms. She visited a private hospital, where her temperature was recorded at 38.5 °C, and received two doses of intravenous antibiotics and nebulizations of salbutamol and budesonide.

The patient's lower limb pain was not accompanied by joint pain, swelling, or skin rashes. In terms of her overall condition, she had decreased oral intake and activity. Her medical history revealed no recent trauma or vigorous exercise prior to the onset of symptoms. There was no ingestion of contaminated food or medications, and she did not have any gastrointestinal symptoms. The patient's family denied any neurological issues or changes in urine frequency, color, or smell. There were no hematological symptoms, such as a bleeding tendency or easy bruising.

The patient had a positive travel history one week prior to the ED visit and a positive family history of a recent upper respiratory tract infection. However, she did not have any known contact with COVID-19 patients. She received vaccinations up to the age of two, follows a regular diet without any known allergies, and has parents who are second-degree relatives with no chronic diseases in the family. She has five healthy siblings, and her developmental milestones are appropriate for her age as she attends kindergarten.

Upon arrival at the ED, the patient presented with stable vital signs. Her blood pressure was 111/67 mmHg, heart rate was 136 beats per minute, respiratory rate was 28 breaths per minute, temperature was 38.4 °C, and oxygen saturation (SpO2) level was 99% on room air. The patient was fully conscious and showed no signs of respiratory distress or marked discoloration. Mild dehydration was observed, and the chest examination revealed clear lungs. The patient's overall condition upon admission was well and alert, with no cyanosis, pallor, or jaundice. As there were no signs of respiratory distress and only mild dehydration, no nasal cannula was attached. However, intravenous fluids were administered to the patient.

The head-ears-eyes-nose-throat (HEENT) assessment revealed normal ears without redness of the tympanic membrane. The throat exhibited congestion without any exudate or pus, and lymphadenopathy was absent. The neuromuscular examination demonstrated normal activity and a full range of motion in all limbs. Muscle tone and power were within normal limits, with intact deep tendon reflexes and no sensory or motor deficits. Meningeal signs such as nuchal rigidity, Kernig's sign, and Brudzinski's sign were all negative. Furthermore, all cranial nerves were intact without any abnormalities, and there were no signs of proximal weakness. During the gait assessment, the patient refused to walk independently but demonstrated weight-bearing ability and took a few steps with assistance from her mother. The Gower sign was negative. The cardiovascular examination revealed normal heart sounds (S1+S2) and intact peripheral pulses, with a capillary refill time (CRT) of less than two seconds. In the chest examination, equal bilateral air entry was observed without any adventitious sounds. The gastrointestinal system assessment showed a soft, non-distended abdomen with no splenomegaly or organomegaly.

**Figure 1:**
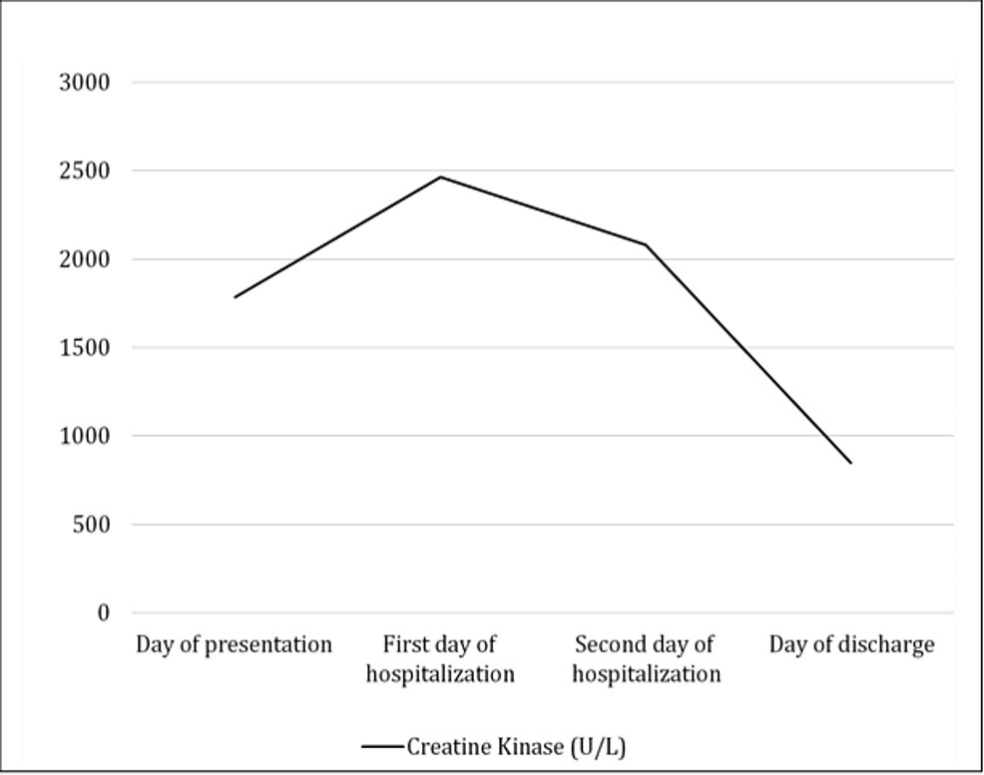
Progression of creatine kinase during hospitalization

On the day of presentation, initial laboratory tests revealed elevated levels of CK (Figure 1). Aspartate aminotransferase (AST) levels were also elevated. Inflammatory markers, including C-reactive protein and procalcitonin, were within the normal range. Lactate dehydrogenase levels were also normal. The complete blood count showed leukopenia with mild neutropenia and lymphopenia. The respiratory virus panel confirmed a positive result for influenza A virus and negative results for COVID-19, influenza B virus, and respiratory syncytial virus. The urine myoglobin test and blood coagulation profile yielded normal findings (Table 1).

**Table 1:** Laboratory evaluations * Lower than normal levels; ^ Higher than normal levels BUN: Blood urea nitrogen; GGT: Gamma-glutamyl transferase; ALT: Alanine transaminase; AST: Aspartate aminotransferase; ALP: Alkaline phosphatase; CK: Creatine kinase; CRP: C-reactive protein; RSV: Respiratory syncytial virus; Flu A: Influenza A; Flu B: Influenza B

Laboratory markers	Investigation	ED visit	Hospitalization			Outpatient appointment
		Day 0	Day 1	Day 2	Day 3	Day 5
Complete blood count	Hemoglobin (g/dl)	12.8	11.6	-	11.4	11.9
	White blood cells (10^9/L)	3.4*	2.9*	-	3.7*	7.7
	Lymphocytes (10^9/L)	1.37*	2.29	-	2.53	3.81
	Neutrophils (10^9/L)	1.30*	0.21*	-	0.56*	2.77
	Platelet (10^9/L)	293	284	-	212	337
Chemistry (blood/serum)	Potassium (mmol/L)	3.8	4.3	4.4	4.1	4.7
	Sodium (mmol/L)	135	138	139	140	139
	Chloride (mmol/L)	100	108	107	107	106
	Creatinine (umol/L)	35*	33*	33*	31*	34*
	BUN (mmol/L)	3.4	2.5*	2.3*	2.4*	4.9
	GGT (U/L)	10	9	8	10	9
	ALT (U/L)	17	21	35^	39^	35^
	AST (U/L)	81^	99^	127^	95^	43
	ALP (U/L)	-	-	132*	126*	155*
	Lactic acid (mmol/L)	0.99	-	-	-	-
	CK (U/L)	1784^	2462^	2080^	848^	
	Total protein (g/L)	68	-	55*	55*	66
Chemistry (urine0	U myoglobin (ng/mL)	-	108	-	-	-
Inflammatory markers	CRP	0.6	-	-	-	-
	Procalcitonin (ug/L)	0.7	-	-	-	-
Respiratory viral panel	COVID-19	Negative				
	Flu A	Positive				
	Flu B	Negative				
	RSV	Negative				

Based on the clinical presentation, laboratory findings, and positive result for influenza A virus, the patient was diagnosed with viral myositis secondary to influenza A infection and was admitted due to dehydration. Treatment with oseltamivir commenced after confirming the positive result for the influenza A virus. The patient was initiated on maintenance intravenous fluids and encouraged to begin oral feeding (Table 2). During her hospital stay, the patient became afebrile, well-hydrated, tolerated oral intake, and was able to walk without pain. After 72 hours, the patient was discharged home with instructions to continue taking oseltamivir orally at a dosage of 45 mg twice daily for five days. Additionally, the mother received instructions on maintaining hydration at home. Two days later, the patient presented for a follow-up appointment at the general pediatric clinic. She appeared healthy and active, able to walk freely without experiencing any pain or limping. Repeat laboratory evaluations showed improvement in CK and all other biomarkers; however, LDH levels were elevated.

**Table 2:** Therapeutic intervention BID: Twice a day

Medications	ED visit	Hospitalization			Outpatient appointment
	Day 0	Day 1	Day 2	Day 3	Day 5
Acetaminophen injection	295 mg	-	-	-	-
Oseltamivir (15 mg/mL)	45 mg BID	45 mg BID	45 mg BID	45 mg BID	-
Supportive measures	Intravenous fluids	Intravenous fluids	Intravenous fluids	Intravenous fluids	-

## Discussion

Viral myositis is a rare but clinically significant condition characterized by muscle inflammation following a recent viral illness, with influenza A or B being the most commonly associated pathogens [6]. The incidence of viral myositis is observed to be higher in cases associated with influenza B (33.9%) compared to influenza A (5.5%) [1,6]. Notably, during influenza epidemics, the rate of viral myositis cases escalates from 0.23 cases per 100,000 to 2.6 cases per 100,000 [7]. The epidemiology of viral myositis shows variations in age distribution and gender predilection. It predominantly affects children and young adults, with a higher prevalence observed in males [4,5]. This gender disparity may be attributed to hormonal and genetic factors, as well as differences in immune responses between males and females.

Regarding the incidence and prevalence of viral myositis in Saudi Arabia, limited data are available. However, a study conducted in the eastern region of Saudi Arabia estimated a prevalence rate of approximately 3.17 per 100,000 pediatric patients under the age of 12 [8]. The pathogenesis is not fully understood. However, animal studies have proposed a potential association between viral myositis and the direct invasion of muscle tissue by the infecting virus. This initial infection causes notable muscle fiber necrosis, leading to an elevation in levels of CK. However, despite these observations, no viruses were detected in biopsy samples, indicating a possible incapacity of the virus to replicate within the muscle tissue. Conversely, in specific cases, influenza virus antigens were identified in the muscle biopsies of patients, suggesting a potential direct viral infection of the muscle. Consequently, there remains uncertainty regarding whether myositis is induced by direct viral activity or immune-mediated mechanisms [10].

Patients with viral myositis present with abrupt bilateral muscle pain and tenderness within one to seven days following the resolution of an upper respiratory or gastrointestinal infection [11]. The pain typically manifests in the lower limbs, particularly in the calf or quadriceps muscles, leading to difficulties in walking and abnormal gait patterns, such as tiptoe walking or a stiff-legged gait [6]. In addition to pain, patients may experience constitutional symptoms such as fever, cough, nausea, vomiting, and other associated symptoms [11]. It is crucial to note that gait changes in patients with myositis are primarily driven by severe pain upon limb movement; thus, it is imperative to ensure normal neurological and strength examinations [11]. Furthermore, a comprehensive medical history should be obtained to investigate any previous similar presentations, recent trauma, or drug use, as well as a family history of metabolic, neuromuscular, and endocrinologic disorders [11].

Viral myositis is a self-limiting disease that can be managed through pain relief, hydration, and regular follow-up. Typically, clinical symptoms demonstrate improvement within three days of the initial onset, and creatine kinase levels tend to decrease within two weeks [6]. Antiviral medications have shown limited efficacy in treating myositis, and the condition often resolves without interventions or complications. However, it is important to note that rare cases may develop symptoms of rhabdomyolysis, such as the presence of tea-colored urine [6]. The diagnosis of viral myositis involves a clinical evaluation and a thorough assessment of symptoms and muscle involvement [2,3]. Supporting the diagnosis and identifying the specific viral agent responsible for the myositis are achieved through laboratory tests, which may include measuring CK levels, conducting a complete blood count, and performing viral serology [2,3]. Laboratory tests can show increased CK, AST, and LDH. A complete blood count and viral serology can help support the diagnosis and identify the viral agent responsible [3].

As a result of the relatively infrequent occurrence of viral myositis, there is a risk that it might not be considered when assessing a pediatric patient presenting with sudden muscle weakness and gait disturbances [7,6]. Physicians often prioritize the investigation of more severe etiologies for muscle weakness, including Guillain-Barré syndrome (GBS), hypokalemia, rhabdomyolysis, and acute post-infectious cerebellar ataxia [4,6]. Other pathologies that present with similar symptoms include dermatomyositis, polymyositis, trauma, or fracture [11].

Guillain-Barré syndrome is a post-infectious disorder that can give rise to ataxia [12]. Patients typically manifest bilateral, ascending, progressive, and symmetrical muscle weakness, primarily originating in the lower limbs, often accompanied by the presence of paresthesia and pain [12]. A prominent characteristic of GBS is the absence or reduction of deep-tendon reflexes. Furthermore, lumbar puncture findings commonly demonstrate elevated levels of protein in the cerebrospinal fluid, while the white blood cell count remains within the normal range [12]. Guillain-Barré syndrome was ruled out in our patient since these clinical findings were not present, and an LP was not performed.

Rhabdomyolysis can manifest as a severe complication of viral myositis, leading to extensive muscle damage and kidney damage or failure [13]. Viral infections such as influenza A and B, Epstein-Barr virus, and enteroviruses have been linked to viral myositis and its progression to rhabdomyolysis [13]. Clinical manifestations often encompass myalgia, weakness, and tea-colored urine. The acute onset occurs within hours following a muscle injury, seizures, or strenuous exercise [14].

Dermatomyositis is an autoimmune disease characterized by muscle inflammation and skin involvement [15]. Unlike viral myositis, it is not caused by a viral infection. This condition typically presents with muscle weakness, particularly in the proximal muscles, and a characteristic skin rash on the face and bending points of the knee and elbow [16]. Another pathology with similar symptoms to viral myositis is polymyositis. Polymyositis is also an autoimmune disorder characterized by persistent muscle inflammation, resulting in a gradual onset of proximal muscle weakness and progressive limitations in everyday activities [17,18]. In contrast, viral myositis has a more acute onset of muscle symptoms that improve once the viral infection resolves [1]. To distinguish the clinical characteristics of viral myositis from other potential pathologies, it is crucial to undertake a comprehensive and thorough medical history and physical examination [6]. This approach will ensure the precise identification and treatment of viral myositis.

## Conclusions

The presence of muscle weakness and abnormal gait in a child during an acute upper respiratory tract infection may raise concerns, but it can often be disregarded when considering potential serious neurological conditions, leading to unnecessary investigations. Conducting a comprehensive neurological examination and identifying signs of myositis, particularly during the appropriate season, can contribute to a more focused and accurate differential diagnosis, thereby facilitating optimal patient care.

## References

[1] Narayanappa G, Nandeesh BN (2021). Infective myositis. Brain Pathol.

[2] Tippett E, Clark R (2013). Benign acute childhood myositis following human parainfluenza virus type-1 infection. Emerg Med Australas.

[3] Magee H, Goldman RD (2017). Viral myositis in children. Can Fam Physician.

[4] Crowley LM, Mazzaccaro RJ, Dunn AL, Bauch SE, Greenberg MR (2021). Don’t forget the flu — determining the etiology of infective myositis in a child: a case report. Clin Pract Cases Emerg Med.

[5] Hu JJ, Kao CL, Lee PI (2004). Clinical features of influenza A and B in children and association with myositis. J Microbiol Immunol Infect.

[6] Hyczko AV, Rohrbaugh MK, Suliman AK, Hackman NM (2021). A crawling case of benign acute childhood myositis. SAGE Open Med Case Rep.

[7] Buss BF, Shinde VM, Safranek TJ, Uyeki TM (2009). Pediatric influenza-associated myositis — Nebraska, 2001-2007. Influenza Other Respir Viruses.

[8] Al-Qahtani MH, Salih AM, Yousef AA (2015). Benign acute childhood myositis in the eastern region of Kingdom of Saudi Arabia: a 5-year experience. J Taibah Univ Med Sci.

[9] Mackay MT, Kornberg AJ, Shield LK (1999). Benign acute childhood myositis: laboratory and clinical features. Neurology.

[10] Koliou M, Hadjiloizou S, Ourani S, Demosthenous A, Hadjidemetriou A (2010). A case of benign acute childhood myositis associated with influenza A (H1N1) virus infection. Clin Microbiol Infect.

[11] Hernández MLP, Latorre JRV, Ortegón-Ochoa S, Naranjo-Medina N, Pacheco B (2019). Viral myositis, a pediatric case report. Arch Argent Pediatr.

[12] van Doorn PA, Ruts L, Jacobs BC (2008). Clinical features, pathogenesis, and treatment of Guillain-Barré syndrome. Lancet Neurol.

[13] Nauss MD, Schmidt EL, Pancioli AM (2009). Viral myositis leading to rhabdomyolysis: a case report and literature review. Am J Emerg Med.

[14] Szugye HS (2020). Pediatric rhabdomyolysis. Pediatr Rev.

[15] Qudsiya Z, Waseem M (2023). Dermatomyositis. https://www.ncbi.nlm.nih.gov/books/NBK558917/.

[16] Agyeman P, Duppenthaler A, Heininger U (2004). Influenza-associated myositis in children. Infection.

[17] Sarwar A, Dydyk AM, Jatwani S (2023). Polymyositis. https://www.ncbi.nlm.nih.gov/books/NBK563129/.

[18] Anić B, Cerovec M (2012). Polymyositis/dermatomyositis — clinical picture and treatment. Reumatizam.

